# In Vitro Anti-Leptospiral Activity of *Phyllanthus amarus* Extracts and Their Combinations with Antibiotics

**DOI:** 10.3390/ijerph18062834

**Published:** 2021-03-10

**Authors:** Che Ain Munirah Ismail, Zakuan Zainy Deris, Ruzilawati Abu Bakar, Nabilah Ismail

**Affiliations:** 1 Department of Medical Microbiology & Parasitology, School of Medical Sciences, Health Campus, Universiti Sains Malaysia, Kubang Kerian, Kelantan 16150, Malaysia; cheainmunirah91@gmail.com (C.A.M.I.); zakuan@usm.my (Z.Z.D.); 2 Hospital Universiti Sains Malaysia, Universiti Sains Malaysia Kampus Kesihatan, Jalan Raja Perempuan Zainab 2, Kota Bharu, Kelantan 16150, Malaysia; ruzila@usm.my; 3 Department of Pharmacology, School of Medical Sciences, Health Campus, Universiti Sains Malaysia, Kubang Kerian, Kelantan 16150, Malaysia

**Keywords:** *Phyllanthus amarus*, *Leptospira*, anti-leptospiral, susceptibility testing, penicillin G, ceftriaxone, doxycycline

## Abstract

Despite modern medicine, there is an increasing trend for cases of the bacterial infection leptospirosis, and this has led to the exploration of alternative medicines from various sources including plants. The aim of this study was to investigate the in vitro anti-leptospiral activity of *Phyllanthus amarus* extracts alone and combined with penicillin G, ceftriaxone, and doxycycline. Antimicrobial susceptibility testing was performed using the microdilution broth technique upon methanol extract (ME), aqueous extract (AE), and antibiotics against the *Leptospira interrogans* serovars Australis, Bataviae, Canicola, and Javanica, to determine minimum inhibitory concentrations (MICs) and minimum bactericidal concentrations (MBCs). The results were analyzed using an ELISA microplate reader combined with microscopic analysis. Synergy testing using a checkerboard assay was performed to determine the fractional inhibitory concentration index values of extracts combined with antibiotics against leptospires. Scanning electron microscopy (SEM) was used to investigate morphological changes of leptospires caused by potential anti-leptospiral agents alone and combined with antibiotics. The MICs and MBCs for *P. amarus* extracts ranged from 100 to 400 µg/mL for AEs and from 400 to 800 µg/mL for MEs. Penicillin G was the most effective anti-leptospiral drug, with MICs and MBCs ranging from <0.01 to 0.78 and <0.01 to 3.13 µg/mL, respectively, followed by ceftriaxone, with both MICs and MBCs ranging from 0.05 to 0.78 µg/mL, and doxycycline, with MICs and MBCs ranging from 0.39 to 3.13 µg/mL and 12.5 to 25 µg/mL, respectively. Combinations of *P. amarus* extracts and antibiotics did not show synergistic effects on all tested *Leptospira* serovars, with some combinations demonstrating antagonistic effects. SEM analysis, however, showed distorted *Leptospira* surfaces. *P. amarus* AE performed better anti-leptospiral activity than *P. amarus* ME. The morphological effects of *P. amarus* extract alone and its combination with antibiotic on *Leptospira* cells revealed promising anti-leptospiral properties.

## 1. Introduction

Leptospirosis is a re-emerging bacterial infection causing serious concerns worldwide [1]. It occurs in urban environments of both industrialized and developing countries, as well as in rural areas [2]. Leptospirosis cases are endemic in tropical and subtropical regions, and become epidemic in monsoon seasons and flooding [3]. The incidence is significantly high in countries with warm climates compared to the temperate regions, causing the long survival rate of leptospires in the warm and humid conditions. The World Health Organization reported that leptospirosis cases commonly occur in the countries of South-East Asia, and their magnitude differs between the countries, depending on attitude and awareness of the public health care decision makers [4,5]. It is estimated that 1.03 million annual cases with 58,999 deaths occur worldwide due to leptospirosis [6].

Infection of leptospirosis ranges from mild febrile illness to severe fatal disease. The early phase of the infection (anicteric form) shows symptoms of fever, headache, chills, nausea, malaise, myalgia, vomiting, and conjunctival hyperemia. The severe form of leptospirosis (icteric form of leptospirosis known as Weil’s disease) [7] includes the symptoms of jaundice, myocarditis, meningitis, renal failure, uveitis, multi organ failure, and lethal pulmonary hemorrhage [8]. The increasing incidence of leptospirosis with high mortality rates underlines the need for research and updated information on its management and treatment [1]. As the available therapies for leptospirosis in modern medicine are limited, the search for alternative medicines to replace synthetic ones has attracted increasing interest in traditional medicines. Along with animals and microorganisms, plant-based natural products used in the medicinal field contain a wide range of bioactive components useful for treating various diseases [9]. However, research on natural products that can be used as anti-leptospiral agents is scarce and needs to be advanced urgently.

*Phyllanthus amarus*, commonly known as black catnip, carry me seed, or windbreaker, found in tropical and subtropical countries worldwide, has been used for treating problems of the stomach, spleen, kidney, liver, and genitourinary system [10]. In Ayurveda, *P. amarus* is used for treating dysentery, genital infections, gonorrhea, and urinary tract infections [11]. *P. amarus* has shown a wide range of antimicrobial activity. Methanol extracts (MEs) of *P. amarus* exert activity against *Shigella* spp., *Vibrio cholera*, *Bacillus subtilis*, *Streptococcus pneumonia, S. mutans*, *S. salivarius*, *S. pyogenes*, *Penicillium* spp., and *Aspergillus niger* [12,13,14,15,16]. Oluwafemi and Debiri found that the activity of MEs against the *Salmonella* serotype Typhi has comparable zone of inhibition to that of ciprofloxacin, a widely used antibiotic for typhoid fever [17]. *P. amarus* also has anti-leptospiral effects, damaging the DNA of the *Leptospira* serovars Icterohaemorrhagiae, Canicola, and Pomona [18]. However, research on the anti-leptospiral activity of *P. amarus* remains limited. Therefore, this study aims to investigate the in vitro anti-leptospiral activity of *Phyllanthus amarus* extracts alone and combined with penicillin G, ceftriaxone, and doxycycline.

## 2. Materials and Methods

### 2.1. Sampling and Plant Extraction

Mature whole *P. amarus* (Schumach. and Thonn.) plants were harvested from Peringat, Kota Bharu, Kelantan, Malaysia, and sent to the Forest Research Institute Malaysia for identification as PID 150616-14 (Supplementary Figure S1). Whole plants were then washed and dried in the shade, powdered, and stored in dry place at room temperature.

A ME was prepared by adding 30 g of plant powder to 250 mL of methanol (Merck, Darmstadt, Germany), with extraction at 56–60 °C for five hours using a Soxhlet apparatus (Electrothermal, Staffordshire, UK) to achieve optimal oil yield from the plant [19], followed by evaporation to dryness using a vacuum rotary evaporator (337 mbar) at 250 rpm and 40 °C (Buchi, Flawil, Switzerland), and then storage in an airtight container at room temperature. An AE was prepared by adding 60 g of plant powder to 200 mL of distilled water and soaking for three days with continuous stirring using a magnetic stirrer (500 rpm) at room temperature (Thermo Fisher Scientific, Shanghai, China) [20]. Concentrated filtered solutions were freeze-dried (Ilshin Lab. Co., Ltd., Gyeonggi-do, Korea) into a powder and stored. Extract stock solutions were prepared by dissolving 8 mg of plant powder in 1 mL of phosphate-buffered saline (PBS; Sigma-Aldrich, St. Louis, MO, USA). A filtered working solution (3200 µg/mL) was used immediately after preparation.

### 2.2. Ellinghausen McCullough Johnson Harris Broth Preparation

Ellinghausen McCullough Johnson Harris (EMJH) broth was prepared by dissolving Difco^TM^ *Leptospira* Medium Base EMJH (Becton, Dickinson and Company, Sparks, MD, USA) in distilled water (2.3 g/L), which was autoclaved (Tomy Kogyo Co., Ltd., Tokyo, Japan) and left to cool. Difco^TM^ *Leptospira* Enrichment EMJH (Becton, Dickinson, and Company, Sparks, MD, USA) (100 mL/L) and filtered (0.22-µm syringe filter; Bioflow, Kuala Lumpur, Malaysia), and 5-fluorouracil (5-FU; Intas Pharmaceuticals Ltd., Ahmedabad, India) (200 µg/mL) was added. The broth was transferred to 15-mL centrifuge tubes (Nest Biotech Co., Ltd., Wuxi, Jiangsu, China).

EMJH agar was prepared by dissolving Difco^TM^ *Leptospira* Medium Base EMJH (2.3 g/L) and Bacto^TM^ Agar (Becton, Dickinson and Company, Sparks, Maryland, USA) (9 g/mL) in distilled water, followed by autoclaving, leaving to cool, and then adding Difco^TM^ *Leptospira* Enrichment EMJH (100 mL/L) supplemented with filtered 5-FU (200 µg/mL). The solution was poured into plastic petri dishes. Both EMJH broth and agar were incubated at 30–35 °C for 24 h for sterility testing and stored in a cold room prior to use.

### 2.3. Antibiotic Solution Preparation

Penicillin G (Karnataka Antibiotics and Pharmaceuticals Limited, Karnataka, India), ceftriaxone (F. Hoffmann-La Roche Ltd., Basel, Switzerland), and doxycycline (Sigma-Aldrich, St. Louis, MO, USA) stock solutions were prepared by dissolving 1 g of powder to 1 mL of sterile distilled water. The working solutions (25 µg/mL) were used immediately after preparation.

### 2.4. Leptospira Inoculum Preparation

Reference strains of the *Leptospira interrogans* serovars Australis, Bataviae, and Canicola were obtained from Universiti Putra Malaysia, and *L. interrogans* serovar Javanica cultures were obtained from the Institute for Medical Research, Malaysia. The strains were subcultured and incubated with shaking (Jeio Tech, Daejeon, Korea) at 30 °C and 40 rpm for seven days. The resultant cultures and were diluted in EMJH broth to an optical density of 0.32 at 420 nm (approximately 1 × 10^8^ colony-forming unit per milliliter (CFU/mL)) by an indirect method using an ultraviolet-visible spectrophotometer (Shimadzu, Kyoto, Japan) and then further serial-diluted to 2 × 10^6^ CFU/mL in EMJH broth [21].

### 2.5. Antimicrobial Susceptibility Testing

The broth microdilution method was used to determine the susceptibility of *Leptospira* species to penicillin, ceftriaxone, and doxycycline. The results were interpreted based on minimum inhibitory concentrations (MICs) and minimum bactericidal concentrations (MBCs) [22]. One hundred microliters of EMJH medium broth was transferred into wells in 96-well plates (Nest Biotech Co., Ltd., Wuxi, Jiangsu, China), except for the first column. One hundred microliters of working extract or antibiotic solutions was added to the first column with a final volume of 200 µL and mixed well. Then, 100 µL from the first column was transferred to the second column with a final volume of 200 µL (two-fold dilution) and mixed well. The steps of two-fold serial dilution were repeated up to the tenth column with a final volume of 200 µL, producing concentration ranges of 0.01–25 µg/mL for the antibiotic solution and 1.56–3200 µg/mL for the extract solution. One hundred microliters from the tenth column was discarded with a final volume of 100 µL for each well. One hundred microliters of *Leptospira* culture was added into the first column until the tenth column with a final volume of 200 µL for each well. Positive (100 µL broth and 100 µL *Leptospira*) and negative control (200 µL broth) wells were included in the eleventh and twelfth columns, respectively. A growth control plate was prepared by replacing *Leptospira* cultures with broth. The plates were incubated at 30 °C and 40 rpm for seven days. The absorbance of the test plate was read by an automatic enzyme-linked immunosorbent assay (ELISA) tray reader (Thermo Labsystems, Helsinki, Finland) at 420 nm. To eliminate interferences, the control absorbance value was subtracted from the total absorbance values, and the pre-incubation absorbance values were subtracted from the post-incubation values. Turbidity and an obvious white dot at the bottom of wells showed *Leptospira* growth, and this was confirmed by examining a loopful of each well’s contents under a dark field microscope (Olympus, Tokyo, Japan) at 200× magnification, zooming in and out [23]. Wells of MICs and the next two dilutions of increased concentrations were proceeded to MBC determination by inoculating a loopful of each well onto the agar EMJH medium and incubating at 30 °C for two to three weeks. Each experiment was triplicated to ensure reliability.

### 2.6. Synergy Testing by Checkerboard Assay

**Figure 1:**
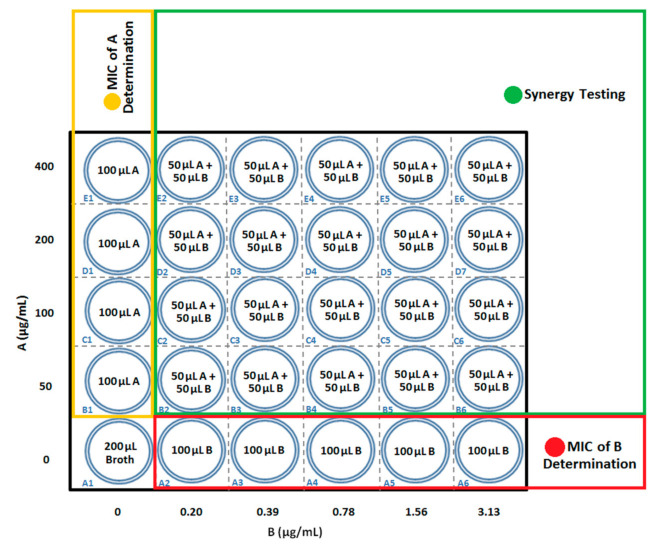
Arrangement of synergy testing in a 96-well plate. A: Extract; B: Antibiotic.

For MIC testing of the extract, 100 µL EMJH broth was added to the first column of a 96-well plate. One hundred microliters of extract was added to E1 well with a final volume of 200 µL. The extract was serially diluted by transferring 100 µL of the extract solution from E1 to the next well by two-fold. Then, 100 μL of *Leptospira* cultures was added to the first column of the 96-well plate (final volume of 200 μL per well). For MIC testing of the antibiotic, 100 µL EMJH broth was added to the first row of the 96-well plate. One hundred microliters of the antibiotic was added to A6 well with a final volume of 200 µL. The antibiotic was serially diluted by transferring 100 µL of the antibiotic solution from A6 to the next well by two-fold. Then, 100 μL *Leptospira* cultures were added to the first row of the 96-well plate (final volume of 200 μL per well). For synergy testing, the two-fold serial dilutions of 50 µL antibiotic and 50 µL extract were prepared and transferred into the second column until the twelfth column. Next, 100 µL of *Leptospira* culture was added into the respective columns (final volume of 200 µL per well) (Figure 1). Control plates were prepared with broth to replace *Leptospira* cultures. The plates were incubated at 30 °C and 40 rpm for seven days. The control absorbance value was subtracted from the total absorbance value determined by an ELISA reader, and the pre-incubation values were subtracted from the post-incubation values. Each of the synergy testing was duplicated. Turbidity and an obvious white dot at the bottom of wells showed *Leptospira* growth, and this was confirmed by examining a loopful of each well’s contents under a dark field microscope (Olympus, Tokyo, Japan) at 200× magnification, zooming in and out [23]. Fractional inhibitory concentration index (FICI) values were calculated for the wells with the lowest concentration showing no visible growth using formulas 1 and 2, and interpreted according to criteria of the American Society of Microbiology and the British Society of Antimicrobial Chemotherapy as follows: synergy (FICI ≤ 0.5), additive (FICI > 0.5–1.0), indifferent (FICI > 1.0–4.0), and antagonistic (FICI > 4.0) [24,25]:

(1) $$\mathrm{FICI}\;\mathrm{of}\;\mathrm{well}=\mathrm{FIC}\;\mathrm{of}\;\mathrm{Antibiotic}+\mathrm{FIC}\;\mathrm{of}\;\mathrm{Extract}=\left(\frac{\mathrm{Concentration}\;\mathrm{of}\;\mathrm{Antibiotic}}{\mathrm{MIC}\;\mathrm{of}\;\mathrm{Antibiotic}}\right)+\left(\frac{\mathrm{Concentration}\;\mathrm{of}\;\mathrm{Extract}}{\mathrm{MIC}\;\mathrm{of}\;\mathrm{Extract}}\right),$$

(2) $$\mathrm{FICI}\;\mathrm{of}\;\mathrm{test}=\frac{\sum \mathrm{FICI}\;\mathrm{of}\;\mathrm{wells}}{\mathrm{numbers}\;\mathrm{of}\;\mathrm{wells}}$$

### 2.7. Scanning Electron Microscope Analysis

The combinations with the lowest FICI values were examined using scanning electron microscopy (SEM)*. Leptospira* cultures (2 × 10^6^ CFU/mL) were incubated at 30 °C and 40 rpm for seven days and treated with the MICs of the extracts and antibiotic solutions separately and combined for 18 h. A negative control (untreated *Leptospira*) sample was prepared. The samples were centrifuged (4560 rpm at 2000 g for 10 min) (Hettich, Westphalia Germany) and the pellets were fixed using McDowell-Trump’s fixative (Electron Microscopy Sciences, Hatfield, PA, USA) at 4 °C for two hours, centrifuged again, washed with 0.1 M PBS (Electron Microscopy Sciences, Hatfield, PA, USA), and fixed using 1% osmium tetroxide (Electron Microscopy Sciences, Hatfield, PA, USA) at 4 °C for one hour. The pellets were then washed with 0.1 M PBS and dehydrated using 50%, 75%, 95%, and 100% acetone (Electron Microscopy Sciences, Hatfield, PA, USA) for ten minutes each. Then, 100% hexamethyldisilazane (Electron Microscopy Sciences, Hatfield, PA, USA) was added to the pellets for 10 min and air-dried overnight. Specimens were then mounted on sample stubs and coated with gold (Electron Microscopy Sciences, Hatfield, PA, USA) for examination with a high-resolution versatile SEM instrument (FEI, Brno, Czech Republic).

### 2.8. Statistical Analysis

The data were analyzed using SPSS software version 26 (IBM, Armonk, NY, USA) and presented as mean ± SD of the triplicates for antimicrobial susceptibility testing and duplicates for synergy testing.

## 3. Results

### 3.1. Antimicrobial Susceptibility Testing

The MICs and MBCs of the extracts and antibiotics are shown in Table 1 and Table 2, respectively. Both *P. amarus* AEs and MEs exhibited anti-leptospiral effects. However, AEs were more effective, with both MICs and MBCs ranging from 100 to 400 µg/mL, whilst MEs ranged from 400 to 800 µg/mL. Penicillin G showed the strongest anti-leptospiral activity, with MICs and MBCs ranging from 0.01 to 0.78 µg/mL and 0.02 to 3.13 µg/mL, respectively, followed by ceftriaxone, with both MICs and MBCs ranging from 0.05 to 0.78 µg/mL, and doxycycline, with MICs and MBCs ranging from 0.39 to 3.13 µg/mL and 12.5 to 25 µg/mL.

### 3.2. Synergy Testing

Table 3, Table 4, Table 5 show the FICI values and antibacterial effects of extracts combined with antibiotics. *P. amarus* AE combined with antibiotics exhibited indifferent effects against *Leptospira* serovars (FICI 1.59–3.41), while combinations of *P. amarus* ME with antibiotics exhibited indifferent or antagonistic activities (FICI 2.51–4.87). *P. amarus* AE combined with doxycycline against *L. interrogans* serovar Australis had the lowest FICI values (1.59), showing indifferent activity. *P. amarus* ME combined with doxycycline against *L. interrogans* serovar Bataviae and penicillin G against *L. interrogans* serovar Canicola had antagonistic effects (FICI 4.22 and 4.87, respectively).

### 3.3. SEM Analysis

**Figure 2:**
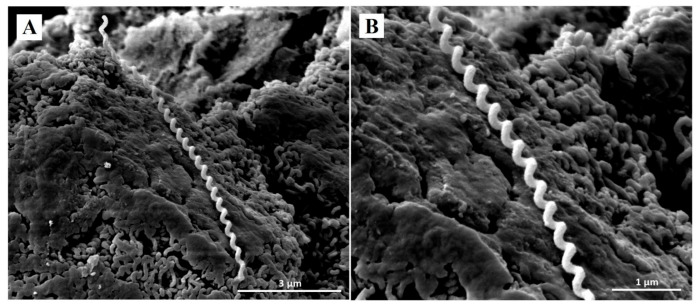
SEM micrographs of normal *L. interrogans* serovar Australis at 30,000× (**A**) and 60,000× (**B**) magnifications with perfect helical-shaped and hooked-end structures.

**Figure 3:**
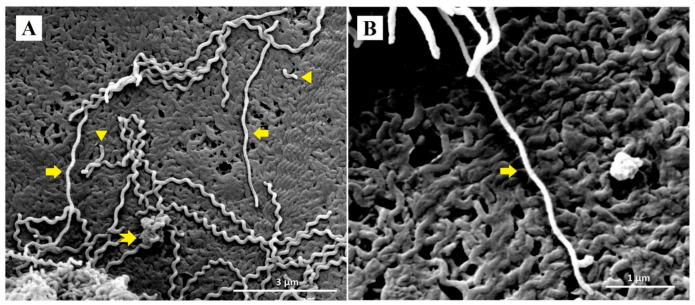
SEM micrographs of *L. interrogans* serovar Australis treated with MIC doxycycline at 30,000× (**A**) and 60,000× (**B**) magnifications. Block arrows: less coiling and thinning in *Leptospira* diameters; notched arrow: blebbing; arrowhead: shortened.

**Figure 4:**
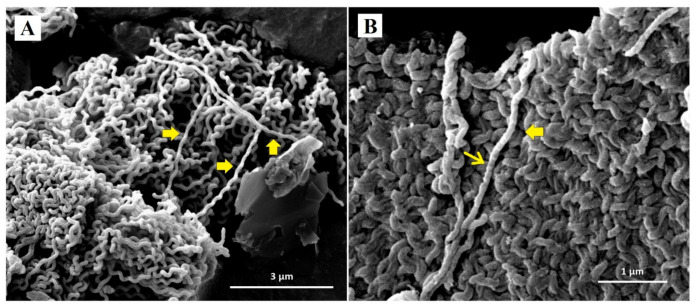
SEM micrographs of *L. interrogans* serovar Australis treated with MIC AE at 30,000× (**A**) and 60,000× (**B**) magnifications. Block arrows: less coiling; arrow: irregular surfaces of *Leptospira* cells.

**Figure 5:**
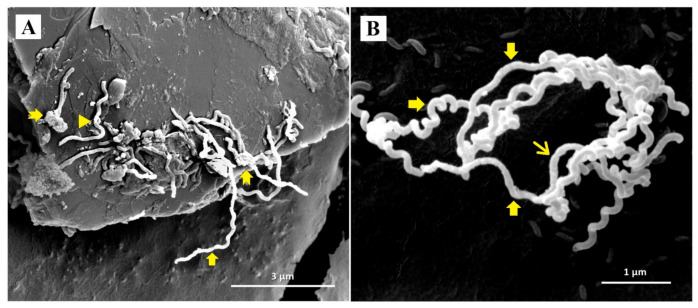
SEM micrographs of *L. interrogans* serovar Australis treated with the MIC of doxycycline combined with the MIC of AE at 30,000× (**A**) and 60,000× (**B**) magnifications. Block arrows: distorted and less coiling; arrow: thinning; arrowhead: shortened; notched arrows: irregular and blebbing appearances.

Representative SEM micrographs revealed notable structural differences between untreated *Leptospira* and *Leptospira* treated with doxycycline and *P. amarus* AE. SEM micrographs of control *Leptospira* cells showed normal spirochaete morphology with helical-shaped and hooked-end structures (Figure 2). *Leptospira* treated with the MIC of doxycycline (0.78 µg/mL) exhibited thinning and less coiling (Figure 3), while *Leptospira* treated with the MIC of *P. amarus* AE (800 µg/mL) exhibited less coiling and irregular surfaces (Figure 4). *Leptospira* treated with *P. amarus* AE combined with doxycycline exhibited severe damage (Figure 5), with thinning, shortening, less and distorted coiling, and irregular surfaces with a blebby appearance.

## 4. Discussion

The results suggest that *P. amarus* AE exhibited stronger anti-leptospiral effects than ME. The MIC ranges of both AE and ME coincided with their MBC ranges, suggesting that the same concentration of *P. amarus* simultaneously inhibits and completely kills the *Leptospira.* Unlike our study, which found that *P. amarus* AE was more effective against *L. interrogans* than ME, other studies have reported that MEs are more effective than AEs against other bacteria, as well as fungi. MEs of aerial parts of the plant have shown more potent inhibitory effects against *Bacillus subtilis*, *Escherichia coli*, *Pseudomonas aeruginosa*, *Staphylococcus aureus*, *Salmonella* serotype Typhi, and *Candida albicans* than AEs [26]. Similarly, Sen and Batra reported that aqueous leaf extracts exhibited minimal activity against *B*. *cereus*, *E. coli*, *S. aureus*, *P*. *aeruginosa*, and *Aspergillus niger*, *A*. *flavus, Fusarium oxysporum*, and *Rhizopus stolonifer* [27]. Conversely, Oladosu et al. found no significant differences between the effects of AEs and MEs against *P. aeruginosa*, *S. saprophyticus*, *S. aureus*, *Klebsiella pneumoniae*, *E. coli*, *Proteus mirabilis*, and *Enterococcus faecalis* [12]. The differences between the antimicrobial effects observed in previous studies may be due to differences in plant extraction parameters, parts of the plant used, and geographical settings. Extracted phytochemicals even vary according to pre- and post-harvest stages [28].

Our preliminary research found differences in anti-leptospiral effects between different extracts. This suggests that the extraction process and choice of solvents play a vital role in extracting components that best inhibit the growth of *Leptospira* or kill it. Previous studies have found slight differences in phytochemical contents between AEs and MEs. Phytochemical analyses of AEs have detected the presence of primary (carbohydrates, proteins, and amino acids) and secondary metabolites (anthraquinone, steroids, including phytosterols and cardiac glycosides, alkaloids, terpenoids, including triterpenes and saponins, phenolic compounds, including flavonoids and lignins, and tannins, specifically phlobatannins) [12,16,17,26,29,30]. Analyses of MEs have revealed the presence of carbohydrates, alkaloids, saponins, terpenoids (triterpenes), steroids (cardiac glycosides), phenolic compounds (flavonoids), and tannins (phlobatannins) [12,16,30,31]. Shirahatti, Syed, and Elgorban investigated the anti-leptospiral effects of three novel biologically active compounds isolated from MEs. Of those, 4-(3-(3,4dimethoxybenzyl)-4-methoxy-2-(methoxymethyl)butyl)-3,6dimethoxybenzene-1,2-diol exhibited the most significant activity against *L. interrogans* serovars Icterohaemorrhagiae, Canicola, Pomona, Autumnalis, Javanica, Pyrogenes, Australis, and Hardjo, followed by 5-(3-(3,4-dimethoxybenzyl)-4-methoxy-2-(methoxymethyl)butyl)4,7-dimethoxybenzo[d][1,3]dioxole and 1-(3-(3,4-dimethoxybenzyl)-4methoxy-2-(methoxymethyl)butyl)-2,3,4,5tetramethoxybenzene [32].

Flavonoids, tannins, terpenoids, alkaloids, lignins, saponins, anthraquinones, and steroids exert antibacterial effects, mainly by disrupting the formation or structure of bacteria cells, causing them to increase their fluidity. These changes lead to an uncontrolled efflux of metabolites, ions, and membrane proteins, causing leakage of cell contents, resulting in cell damage, lysis, and eventually cell death. Flavonoids, saponins, tannins, and anthraquinones disrupt the microbial cell wall by increasing its permeability, leading to cytoplasm leakage. Terpenoids, alkaloids, tannins, and anthraquinones inhibit biofilm formation, thus reducing resistance to antibacterial agents and environmental conditions. Terpenoids, alkaloids, saponins, and tannins inhibit microbial growth by disrupting microbial cell physiology and metabolism, including the inhibition of protein, nucleic acid, and adenosine triphosphate synthesis [33,34,35,36,37,38,39,40]. In reference to mechanisms of action of phytochemicals found in both AEs and MEs against microorganisms, the actions might be applied to *Leptospira* as well. The effects observed in this study might be exerted not only by their major components separately, but also by their synergism.

Our study found that the tested antibiotics were more effective in inhibiting *Leptospira* growth than *P. amarus* extracts. This can be explained by the fact that they are in pure form, whereas crude plant extracts contain substances that may not be particularly effective in inhibiting *Leptospira* growth or killing the bacteria. *Leptospira* serovars were more susceptible to beta-lactam antibiotics, primarily penicillin G and to a lesser degree ceftriaxone, and less susceptible to doxycycline. Similar findings have been reported by previous studies [41,42,43]. In our study, doxycycline was most effective against *L. interrogans* serovar Bataviae, followed by serovars Australis, Canicola, and Javanica. A higher susceptibility of *L. interrogans* serovar Bataviae than serovar Canicola to doxycycline has also been observed in previous studies [43,44,45]. The high MBC ranges of doxycycline observed in this study suggest that higher concentration is required to kill the *Leptospira* completely.

In this study, ceftriaxone was most effective against *L. interrogans* serovar Bataviae, followed by serovars Australis, Canicola, and Javanica. Its MICs and MBCs against *L. interrogans* serovars Bataviae and Javanica coincided, suggesting that it inhibits and kills the *Leptospira* at the same concentrations. Penicillin G was found to be effective against all *Leptospira* spp. and most effective against *L. interrogans* serovar Australis, followed by serovars Bataviae, Canicola, and Javanica. In line with our findings, previous studies have reported that *L. interrogans* serovar Bataviae is more susceptible to penicillin G, as well as to doxycycline, than *L. interrogans* serovar Canicola [43,44,45].

Our study found indifferent inhibitory effects of combinations of antibiotics and extracts against *Leptospira* serovars, except for the antagonistic effect of *P. amarus* ME combined with doxycycline on *L. interrogans* serovar Bataviae and the antagonistic effect of *P. amarus* ME combined with penicillin G on *L. interrogans* serovar Canicola. The lowest FICI value was that of *P. amarus* AE combined with doxycycline against *L. interrogans* serovar Australis, which is close to additive effect values (>0.5–1.0), suggesting that *P. amarus* extracts could have stronger effects when in a purified form. There are limited data on the synergistic effects of plant extracts against *Leptospira* spp. and no data on *P. amarus* extracts. The only study on synergistic effects of plant extracts against *Leptospira* spp. found that purified xanthone γ-mangostin from *Garcinia mangostana* combined with penicillin G exhibited synergistic activity against *L. interrogans* serovars Autumnalis, Bataviae, and Javanica, no interaction activity against *L. biflexa* serovar Patoc, and antagonistic activity against *L. interrogans* serovar Saigon [46].

In this study, SEM revealed that doxycycline and AE alone, and their combination, caused damage to *L. interrogans* serovar Australis. Cell bulging or blebbing is a known effect of doxycycline. Kersten et al. and Meriläinen et al. reported similar morphological distortions of the spirochaete *Borrelia burgdorferi* when exposed to doxycycline [47,48]. Bacterial blebbing, which is one of the factors leading to cell lysis, is caused by physiological stress and turgor pressure, metabolite depletion, cell ageing, exposure to antibiotics, and pH changes that disturb the cell envelope [49,50]. In living cells, cell blebs transport microbial virulence factors and are involved in cell communication and division [49]. The mechanism of action of doxycycline against *L. interrogans* serovar Australis is associated with thinning, shortening, bleb formation, and reduced coiling. To our knowledge, this is the first study to investigate the effects of *P. amarus* extracts on bacterial morphology. The observed distorted *Leptospira* surface morphology could be due to phytochemical components of *P. amarus* extracts that mainly exert effects on the cellular membranes, which can be further analyzed in the future research. Considering the synergy testing and SEM results, *P. amarus* AE combined with doxycycline causes severe damage to *Leptospira* cells but with no enhancement on its inhibiting and killing abilities on *Leptospira*. Further evaluation of internal structural changes of leptospiral cells by transmission electron microscopy analyses is important to confirm anti-leptospiral activities of phytochemicals in *P. amarus.*

## 5. Conclusions

*P. amarus* showed promising anti-leptospiral properties, with AEs yielding better results than MEs. All tested *Leptospira* serovars were more susceptible to penicillin G than to the other tested antibiotics. Synergy testing revealed moderate effects, indicating a need for improvement in this area. However, *P. amarus* AE combined with doxycycline caused significant damage to *Leptospira* cell morphology, further suggesting the potential of *P. amarus* as an anti-leptospiral agent. Further evaluation of *P. amarus* in animal models study is needed, as they have potential anti-leptospiral property, as well as to investigate the whole effects and mechanisms on the subjects including its toxicity.

## Figures and Tables

**Table 1:** MIC and MBC values (mean ± SD) of *P. amarus* extracts on *Leptospira* serovars in triplicate.

*Leptospira* Serovars	AE (µg/mL)		ME (µg/mL)	
	MIC	MBC	MIC	MBC
*L. interrogans* serovar Australis	400 ± 0.0	400 ± 0.0	800 ± 0.0	800 ± 0.0
*L. interrogans* serovar Bataviae	100 ± 0.0	100 ± 0.0	400 ± 0.0	400 ± 0.0
*L. interrogans* serovar Caniola	100 ± 0.0	100 ± 0.0	400 ± 0.0	400 ± 0.0
*L. interrogans* serovar Javanica	200 ± 0.0	200 ± 0.0	800 ± 0.0	800 ± 0.0

AE: Aqueous Extract; ME: Methanol Extract; MIC: Minimum Inhibitory Concentration; MBC: Minimum Bactericidal Concentration.

**Table 2:** MIC and MBC values (mean ± SD) of antibiotics on *Leptospira* serovars in triplicate.

*Leptospira* Serovars	Doxycycline (µg/mL)		Penicillin G (µg/mL)		Ceftriaxone (µg/mL)	
	MIC	MBC	MIC	MBC	MIC	MBC
*L. interrogans* serovar Australis	0.78 ± 0.00	12.50 ± 0.00	<0.01 ± 0.00	<0.01 ± 0.00	0.20 ± 0.00	0.39 ± 0.00
*L. interrogans* serovar Bataviae	0.39 ± 0.00	12.50 ± 0.00	<0.01 ± 0.00	0.05 ± 0.00	0.05 ± 0.00	0.05 ± 0.00
*L. interrogans* serovar Canicola	1.56 ± 0.00	12.50 ± 0.00	0.10 ± 0.00	0.20 ± 0.00	0.20 ± 0.00	0.39 ± 0.00
*L. interrogans* serovar Javanica	3.13 ± 0.00	25.00 ± 0.00	0.78 ± 0.00	3.13 ± 0.00	0.78 ± 0.00	0.78 ± 0.00

MIC: Minimum Inhibitory Concentration; MBC: Minimum Bactericidal Concentration.

**Table 3:** FICI values of AE and ME in combination with doxycycline against *Leptospira* serovars by checkerboard assays and the activities. The test was duplicated and reported in mean ± SD.

*Leptospira* Serovars	Combination of AE and Doxycycline				Combination of ME and Doxycycline			
	MIC AE (µg/mL)	MIC DOX (µg/mL)	FICI	Activity	MIC ME (µg/mL)	MIC DOX (µg/mL)	FICI	Activity
*L. interrogans* serovar Australis	800 ± 0.00	0.78 ± 0.00	1.59 ± 0.00	Indifferent	800 ± 0.00	0.39 ± 0.00	2.82 ± 0.00	Indifferent
*L. interrogans* serovar Bataviae	50 ± 0.00	0.78 ± 0.00	3.09 ± 0.00	Indifferent	200 ± 0.00	0.39 ± 0.00	4.22 ± 0.00	Antagonistic
*L. interrogans* serovar Canicola	100 ± 0.00	1.56 ± 0.00	2.09 ± 0.00	Indifferent	400 ± 0.00	1.56 ± 0.00	2.65 ± 0.00	Indifferent
*L. interrogans* serovar Javanica	100 ± 0.00	1.56 ± 0.00	2.46 ± 0.00	Indifferent	400 ± 0.00	0.78 ± 0.00	3.58 ± 0.00	Indifferent

AE: Aqueous Extract; ME: Methanol Extract; DOX: Doxycycline; MIC: Minimum Inhibitory Concentration.

**Table 4:** FICI values of AE and ME in combination with penicillin G against *Leptospira* serovars by checkerboard assays and their activities. The test was duplicated and reported in mean ± SD.

*Leptospira* Serovars	Combination of AE and Penicillin G				Combination of ME and Penicillin G			
	MIC AE (µg/mL)	MIC PEN (µg/mL)	FICI	Activity	MIC ME (µg/mL)	MIC PEN (µg/mL)	FICI	Activity
*L. interrogans* serovar Australis	200 ± 0.00	0.02 ± 0.00	2.92 ± 0.00	Indifferent	800 ± 0.00	0.01 ± 0.00	3.32 ± 0.00	Indifferent
*L. interrogans* serovar Bataviae	50 ± 0.00	0.02 ± 0.00	3.41 ± 0.00	Indifferent	400 ± 0.00	0.01 ± 0.00	2.95 ± 0.00	Indifferent
*L. interrogans* serovar Canicola	100 ± 0.00	0.05 ± 0.00	2.42 ± 0.00	Indifferent	400 ± 0.00	0.05 ± 0.00	4.87 ± 0.00	Antagonistic
*L. interrogans* serovar Javanica	100 ± 0.00	0.78 ± 0.00	2.61 ± 0.00	Indifferent	400 ± 0.00	0.78 ± 0.00	3.38 ± 0.00	Indifferent

AE: Aqueous Extract; ME: Methanol Extract; PEN: Penicillin G; MIC: Minimum Inhibitory Concentration.

**Table 5:** FICI values of AE and ME in combination with ceftriaxone against *Leptospira* serovars by checkerboard assays and their activities. The test was duplicated and reported in mean ± SD.

*Leptospira* Serovars	Combination of AE and Ceftriaxone				Combination of ME and Ceftriaxone			
	MIC AE (µg/mL)	MIC CEF (µg/mL)	FICI	Activity	MIC ME (µg/mL)	MIC CEF (µg/mL)	FICI	Activity
*L. interrogans* serovar Australis	400 ± 0.00	0.10 ± 0.00	2.39 ± 0.00	Indifferent	400 ± 0.00	0.20 ± 0.00	3.19 ± 0.00	Indifferent
*L. interrogans* serovar Bataviae	100 ± 0.00	0.05 ± 0.00	2.24 ± 0.00	Indifferent	400 ± 0.00	0.02 ± 0.00	2.51 ± 0.00	Indifferent
*L. interrogans* serovar Canicola	100 ± 0.00	0.20 ± 0.00	2.36 ± 0.00	Indifferent	100 ± 0.00	0.10 ± 0.00	3.22 ± 0.00	Indifferent
*L. interrogans* serovar Javanica	200 ± 0.00	1.56 ± 0.00	2.40 ± 0.00	Indifferent	800 ± 0.00	1.56 ± 0.00	2.75 ± 0.00	Indifferent

AE: Aqueous Extract; ME: Methanol Extract; CEF: Ceftriaxone; MIC: Minimum Inhibitory Concentration.

## Data Availability

The data that support the findings of this study are available from the corresponding author, upon reasonable request.

## Supplementary Materials

The following are available online at https://www.mdpi.com/1660-4601/18/6/2834/s1, Figure S1: Plant identification.
