# Supplementary material for: In Vitro Anti-Leptospiral Activity of Phyllanthus amarus Extracts and Their Combinations with Antibiotics
Source: Int J Environ Res Public Health. 2021 Mar 10;18(6):2834. doi: 10.3390/ijerph18062834 (PMC7998951; doi:10.3390/ijerph18062834)
Supplement: Supplementary file 1 [file ijerph-18-02834-s001.pdf]

Supplementary Figure S1. Plant Identification.

INSTITUT PENYELIDIKAN PERHUTANAN MALAYSIA
Forest Research Institute Malaysia (FRIM)
52109 Kepong, Selangor Darul Ehsan
Tel : 603-6279 7000 Fax: 603-6273 1314
Website : www.frim.gov.my

Ruj Kami: FRIM700-1/1/1(103) Tarikh: 28/06/2016

Che Ain Munirah binti Ismail
PT 546, Rumah Kedai Perinkat
Perinkat, 16400 Kota Bharu
KELANTAN DARUL NAIM

Puan,

MENGENALPASTI NAMA SAINTIFIK/BOTANI DAUN TUMBUHAN

Merujuk kepada sampel daun tumbuhan yang diterima pada 7 Jun 2016 mengenai perkara di atas, pihak Institut ini telah mengenalpasti sampel daun tumbuhan ini seperti berikut:

No. Sampel	Jenis Sampel	Famili	Nama Saintifik/Botani	Nama Tempatan
PID 150616-14	Daun	Phyllanthaceae	*Phyllanthus amarus* Schumach. & Thonn.	Dukong anak

2. Bayaran perkhidmatan termasuk bayaran GST yang dikenakan adalah RM106.00 (Ringgit Malaysia: satu ratus enam sahaja). Bersama ini disertakan salinan asal resit yang bernombor 7161632 untuk rujukan dan simpanan puan.

Sekian, terima kasih.

"BERKHIDMAT UNTUK NEGARA"

Saya yang menurut perintah,

(DR RICHARD CHUNG CHENG KONG)
Pegawai Penyelidik Kanan
Bahagian Biodiversiti Hutan
b.p. Ketua Pengarah FRIM

No Dokumen: 10190539

Intertek
